# Visit-to-Visit Glucose Variability Predicts the Development of End-Stage Renal Disease in Type 2 Diabetes: 10-Year Follow-Up of Taiwan Diabetes Study

**DOI:** 10.1097/MD.0000000000001804

**Published:** 2015-11-06

**Authors:** Ya-Fei Yang, Tsai-Chung Li, Chia-Ing Li, Chiu-Shong Liu, Wen-Yuan Lin, Sing-Yu Yang, Jen-Huai Chiang, Chiu-Ching Huang, Fung-Chang Sung, Cheng-Chieh Lin

**Affiliations:** From the Division of Nephrology (Y-FY, C-CH), China Medical University Hospital; School of Medicine (Y-FY, C-IL, C-SL, W-YL, C-CH, C-CL), College of Medicine, China Medical University; Department of Public Health (Y-FY, F-CS), China Medical University; Institute of Biostatistics (T-CL, S-YY), College of Public Health, China Medical University; Department of Healthcare Administration (T-CL), College of Medical and Health Science, Asia University; Department of Medical Research (C-IL, C-SL, C-CL), China Medical University Hospital; Department of Family Medicine (C-SL, W-YL, C-CL), China Medical University Hospital; Management Office for Health Data (J-HC, F-CS), China Medical University Hospital; and Research Center for Chinese Medicine & Accupuncture (J-HC), China Medical University, Taichung, Taiwan.

## Abstract

The purpose of this study was to examine the association of glucose variability using coefficient of variation of fasting plasma glucose (FPG-CV) and coefficient of variation of glycated hemoglobin (HbA_1c_-CV) to end-stage renal disease (ESRD) in 31,841 Chinese patients with type 2 diabetes.

Patients with type 2 diabetes enrolled in National Diabetes Care Management Program, aged ≧30 years, and free of ESRD (n = 31,841) in January 1, 2002 to December 31, 2004 were included. Extended Cox proportional hazards regression models with competing risk of all-cause mortality were used to evaluate risk factors on ESRD incidence. Patients were followed till 2012.

After a median follow-up period of 8.23 years, 1642 patients developed ESRD, giving a crude incidence rate of 6.27/1000 person-years (6.36 for men, 6.19 for women). After the multivariate adjustment, both FPG-CV and HbA_1c_-CV were independent predictors of ESRD with corresponding hazard ratios of 1.20 (95% confidence interval [CI] 1.01, 1.41), 1.24 (95% CI 1.05, 1.46) in HbA_1c_-CV from fourth to fifth quintile and 1.23 (95% CI 1.03, 1.47) in FPG-CV from fifth quintile.

One-year visit-to-visit glucose variability expressed by FPG-CV and HbA_1c_-CV predicted development of ESRD in patients with type 2 diabetes, suggesting therapeutic strategies toward a goal to minimize glucose fluctuation.

## INTRODUCTION

Diabetes mellitus (DM) ranks as a leading cause of end-stage renal disease (ESRD) in many developed and developing countries.^1^ Controlling glucose had been proposed to be an important way to control diabetic nephropathy. However, assessing glucose control in diabetes can be challenging. Glucose was usually measured by fasting glucose, postprandial glucose, or 3-month average glucose, glycosylated hemoglobin (HbA_1c_) level.^2^ Several studies have revealed the importance of time-averaged mean levels of glycemia, measured by HbA_1c_, and is currently considered the “gold standard” of glycemic control in reducing diabetes-related complications.^3–5^ However, randomized controlled trials such as Action to Control Cardiovascular Risk in Diabetes (ACCORD),^6^ the Action in Diabetes and Vascular Disease (ADVANCE),^7^ and the Veterans’ Administration Diabetes Trial (VADT)^8^ reported that lowering blood glucose did not appreciably reduce ESRD incidence and incidence of serum creatinine doubling or 20 mL/min per 1.73 m^2^ decrease in estimated glomerular filtration rate (eGFR) decline in ACCORD study,^6^ risk of the need for renal-replacement therapy or death from renal causes in ADVANCE study,^7^ and mean GFR decline and incidence of severe renal changes, defined as GFR < 15 mL/min in VADT study.^8^ Such findings may be partly due to the fact that these studies used HbA_1c_ as marker of glucose control, which failed to reflect glucose variability and risk associated with extreme glucose change over a long period of time.^9^ These findings have raised the concern that glucose variability, irrespective of the magnitude of hyperglycemia, may confer an additional risk for the development of micro- and macrovascular diabetic complications.^3,4,9^

Among type 2 DM studies, glucose variability showed a positive association with the development of progression of diabetic retinopathy, cardiovascular events, and mortality.^10^ With regard to nephropathy, HbA_1c_ variability was reported a significant predictor of microalbuminuria, independent of the mean HbA_1c_.^11^ Lin et al reported that not only coefficient of variation in HbA_1c_ (HbA_1c_-CV), but also coefficient of variation in fasting plasma glucose (FPG-CV) had strong association with diabetic nephropathy.^12^ Although fasting plasma glucose and HbA_1c_ were widely used to monitor glucose level, only one study has explored the association between standard deviation of HbA_1c_ (HbA_1c_-SD) and ESRD.^13^ More data are required to characterize the effect that the glucose variation exerts on ESRD. This study used the National Diabetes Care Management Program (NDCMP) registration in Taiwan to test whether glucose variation, measured by FPG-CV or HbA_1c_-CV, is associated with ESRD in type 2 diabetes.

## METHODS

### Study Population and Data Sources

NDCMP is a case management program which enrolled patients with diabetes in medical center, regional hospital, and local clinic nationwide in Taiwan since 2002. DM was clinically diagnosed based on American Diabetes Association criteria (International Classification of Disease, 9th Revision, Clinical Modification [ICD-9-CM] diagnosis code 250), and patients were recruited without restriction for antidiabetes medication. A total of 63,084 ethnic Chinese patients diagnosed with type 2 diabetes were enrolled from 2002 to 2004. Date of entry into NDCMP was defined as index date. We excluded patients with type 1 diabetes (ICD-9-CM code 250.x1/x3), gestational diabetes (ICD-9-CM code 648.83), ESRD at baseline (ICD-9-CM code V45.1 with catastrophic illness identification), being followed up <1 year and persons <30 years of age (n = 3942). Enrollees of NDCMP program have to complete comprehensive evaluation, including demographic data, medical and drug history, blood pressure, body weight, height, waist circumference, and fundoscopy. After 12 hours of overnight fasting, blood was drawn from an antecubital vein and sent for analysis within 4 hours postcollection. Biochemical data including urea nitrogen, creatinine, total cholesterol, triglyceride, alanine transaminase, high-density lipoprotein, low-density lipoprotein, and HbA1c were examined at baseline. Patients were followed up every 3 to 6 months and received blood tests during follow-up visits. This study was approved by the China Medical University Hospital Ethical Review Board (DMR100-IRB-292).

The Taiwan government launched the National Health Insurance (NHI) program in 1995. Ninety-nine percent of Taiwan populations were enrolled in the program, and the proportion of withdrawing from NHI is very low.^14^ Patient demographics, diagnoses, and prescriptions in hospital and outpatient claims were recorded. Claims data are randomly audited by the NHI Bureau. This study used NHI data sets for inpatient care by admission and outpatient visits during 2001 to 2004 to identify baseline comorbidity. Individuals in Taiwan carry unique personal identification numbers (PIN). All NHI and NDCMP data sets can be interlinked with the PIN. For security and privacy purposes, patient identity data are scrambled cryptographically by the NHI Research Database. We followed from 1 year after index date until ESRD, death, or withdrawal from NHI.

### Outcome and Comorbidity Ascertainment

The primary outcome measure, ESRD, was determined by catastrophic illness certification (ICD-9-CM code 585 with V45.1) from the registry for catastrophic illness database of NHI program from 2002 to 2012. The catastrophic illness certification was issued by a nephrologist and confirmed by another nephrologist. We searched all ESRD incident events by excluding those who had ESRD events before the index date. To rule out the possibility of cause-and-effect, those who had ESRD incident events within 1 year of index date were also excluded.

Ten chronic conditions were tabulated for 12 months before enrollment by using NDCMP data set as well as outpatient and inpatient claims data: morbid obesity (body mass index [BMI] ≥27 kg/m^2^), albuminuria (ICD-9-CM code 719.0 or urinary albumin-to-creatinine ratio ≥30 mg/g creatinine), coronary artery disease (ICD-9-CM codes 410–413, 414.01–414.05, 414.8, and 414.9), congestive heart failure (ICD-9-CM codes 428, 398.91, and 402.x1), cancer (ICD-9-CM codes 140–149, 150–159, 160–165, 170–175, 179–189, 190–199, 200, 202, 203, 210–213, 215–229, 235–239, 654.1, 654.10, 654.11, 654.12, 654.13, and 654.14), hyperlipidemia (ICD-9-CM code 272), atrial fibrillation (ICD-9-CM code 427.31), hypertension (ICD-9-CM codes 401–405), chronic hepatitis (ICD-9-CM codes 571, 572.2, 572.3, 572.8, 573.1, 573.2, 573.3, 573.8, and 573.9), chronic obstructive pulmonary disease (ICD-9-CM codes 490, 491–495, and 496).

### Statistical Analysis

For each individual patient, FPG-CV and HbA_1c_-CV were computed using the measurements of outpatient visits within the first year of index date. These 2 measures were calculated only for those who had >2 FPG and HbA_1c_ measurements within the first year. The CV value was divided by the square root of the ratio of total visits divided by total visits minus 1 for the adjustment of variation in the number of visits among individual patients.^15^ FPG-CV and HbA_1c_-CV were categorized into 5 classes according to quintiles. We performed sensitivity analysis by classifying patients into 10 subgroups according to deciles of FPG-CV to assess the impact of different threshold of FPG-CV on our findings. Kaplan–Meier curve for cumulative incidence was generated. We adopted extended Cox proportional hazards models of Lunn–McNeil approach on ESRD incidence by considering competing risk of all-cause mortality. Lunn–McNeil approach fits a proportional subdistribution hazards regression model on cause-specific hazards of each competing risk.^16^ Hazard ratios (HRs) and their 95% confidence intervals (CIs) were reported with adjustment for age, sex, and multiple variables. We considered 2 multivariate models. The first multivariate model adjusted for age (continuous) and sex (female/male). The second one further adjusted for tobacco use (yes/no), alcohol use (yes/no), duration of diabetes (continuous, years), type of hypoglycemic drug (diet or exercise, 1 oral hypoglycemic drug, 2 oral hypoglycemic drugs, 3 oral hypoglycemic drugs, >3 oral hypoglycemic drugs, insulin only, insulin, and oral hypoglycemic drug), antihypertensive treatment (yes/no), obesity (BMI ≥27 kg/m^2^), coronary artery disease (yes/no), congestive heart failure (yes/no), cancer (yes/no), hyperlipidemia (yes/no), hypertension (yes/no), atrial fibrillation (yes/no), chronic hepatitis (yes/no), chronic obstructive pulmonary disease (yes/no), mean values of the first year follow-up of fasting glucose (continuous, mg/dL) and HbA1c (continuous, %), and eGFR (continuous, mL/min/1.73 m^2^). We assessed interaction of FPG-CV and HbA_1c_ by adding their product terms into the full multivariate model with the use of likelihood ratio test for statistical significance. The level of significance was set at 0.05 and all *P* values were 2-tailed. We performed all analyses using the SAS statistical package for Windows (Version 9.4, SAS, Cary, NC).

## RESULTS

**FIGURE 1:**
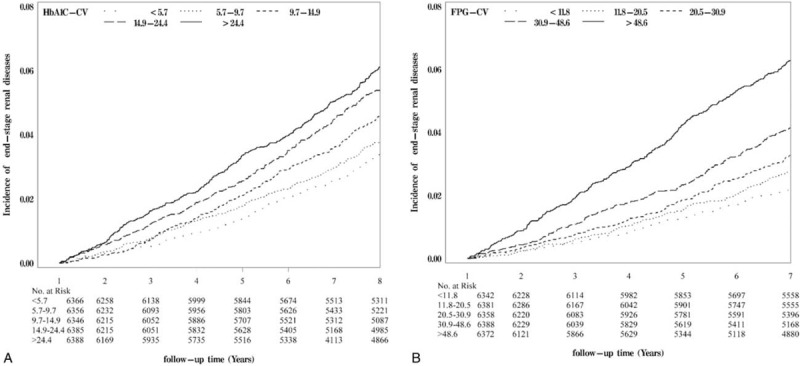
Risks of ESRD for (A) HbA1c-CV and (B) FPG-CV. Log-rank test, all *P* < 0.001. ESRD = end-stage renal diseases.

During January 1, 2002 and December 31, 2004, 63,084 individuals were enrolled in the NDCMP. Twenty-nine thousand eight hundred twenty-two individuals were excluded for age <30 years, with type 1 diabetes, without >2 records of HbA_1c_ or FPG measurements, or being followed up <1 year. One thousand four hundred twenty-one individuals were further excluded because of lacking sociodemographic, lifestyle, drug, or comorbidity information. A total of 31,841 individuals were included for analysis, and 1642 incident ESRD cases were identified during follow-up period with cumulative incidence rates of 4.72%, 6.03%, and 15.97% for patients with normal urinary albumin-to-creatinine ratio, microalbuminuria, and macroalbuminuria and of 1.90%, 2.53%, 9.68%, and 43.15% for patients with eGFR ≥90, 60 to 89, 30 to 59, and <30 mL/min/1.73 m^2^. We compared baseline characteristic between patients included and those excluded by using standardized mean differences. Except for no medication slightly >0.1 SD (0.17), the standardized mean differences for the other variables were <0.1 SD, indicating a negligible difference in means or proportions between groups. In our cohort, the median numbers of FPG and HbA_1C_ measurements within 1 year were both 3 tests and both 25th and 75th percentiles were 2 and 4 tests, respectively. The mean age is 60.94 ± 11.16 years with lowest in the highest quintile in both FPG-CV and HbA_1C_-CV. In the highest quintile of FPG-CV, 17.75% use tobacco, 9.15% drink alcohol, which is highest among other quintiles and is similar to 18.07% and 9.97% in the HbA_1C_-CV group. The highest quintile of FPG-CV and HbA_1C_-CV were not with highest burden of comorbidity. The highest quintile of FPG-CV had the lowest mean BMI and prevalence of hyperlipidemia and hypertension. The highest quintile of HbA_1C_-CV had the lowest mean BMI and prevalence of coronary artery disease, hyperlipidemia, hypertension, and hepatitis. Tables 1 and 2 show baseline sociodemographic and clinical factors in subjects grouped according to quartiles of FPG-CV and HbA_1c_ levels. Patients reaching ESRD have higher mean age, longer diabetes duration, higher prevalence of hypertension drug treatment, congestive heart failure, hyperlipidemia, hypertension, chronic obstructive pulmonary disease, stroke, and hypoglycemia, and lower prevalence of chronic hepatitis. After an average of 8.23 years of follow-up, 1642 patients developed ESRD, giving a crude incidence rate of 6.27/1000 person-years (6.36 for men, 6.19 for women); 6491 died, with a mortality rate of 24.77/1000 person-years (28.82 men, 21.38 women). Figure 1 presents the Kaplan–Meier cumulative risk for ESRD within subgroups defined by FPG-CV and HbA_1c_-CV. Patients with FPG-CV > 48.6% faced higher risk (log-rank test *P* < 0.001, Fig. 1A) similar to those with HbA_1c_-CV > 24.4% (log-rank test *P* < 0.001, Fig. 1B).

**TABLE 1:**
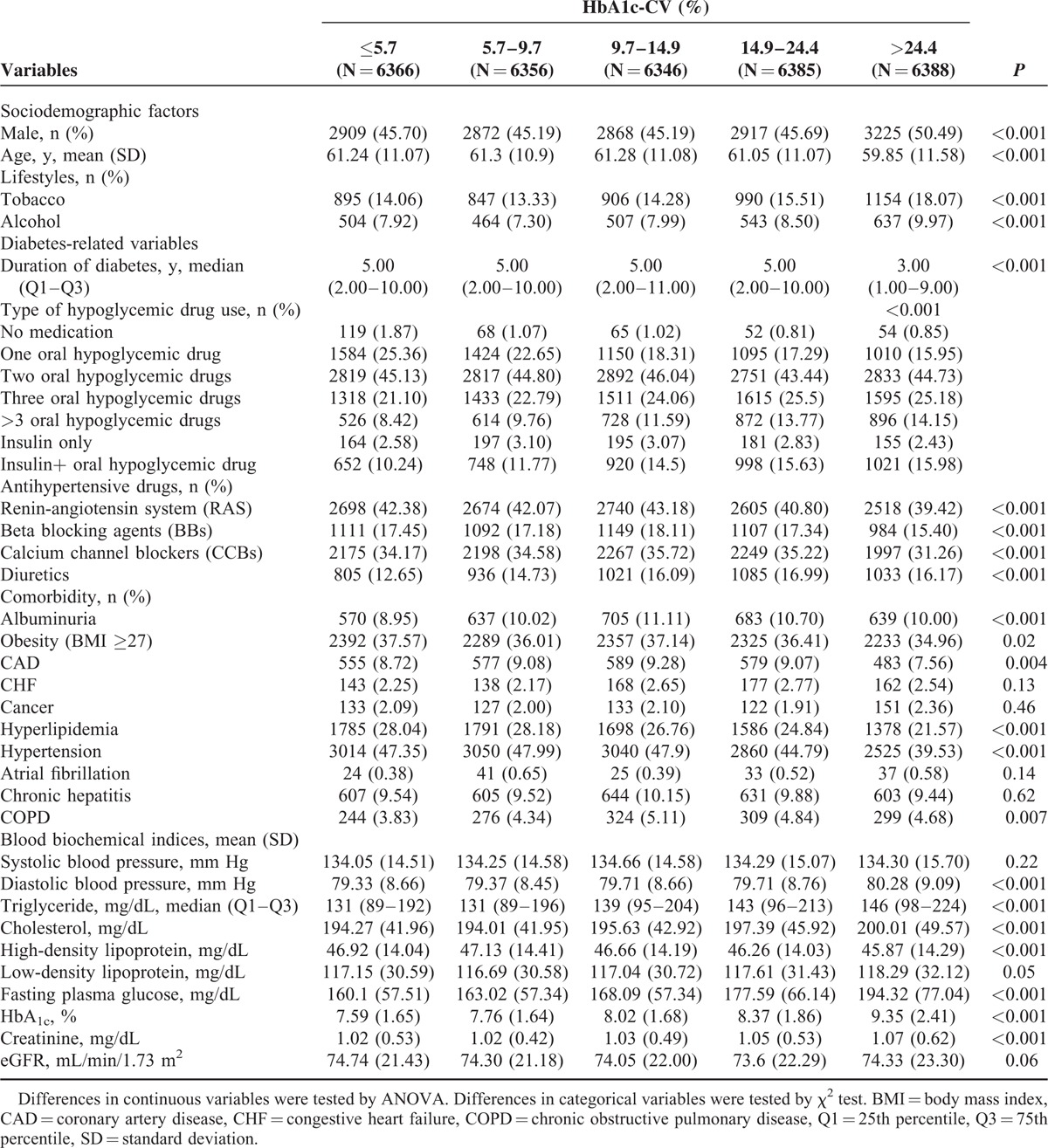
Comparisons of Baseline Sociodemographic Factors, Lifestyle Behaviors, Diabetes-Related Variables, Drug-Related Variables, and Comorbidity According to Quintiles of HbA1c-CV in Patients With Type 2 Diabetes Enrolled in the NDCMP, Taiwan (n = 31,841)

**TABLE 2:**
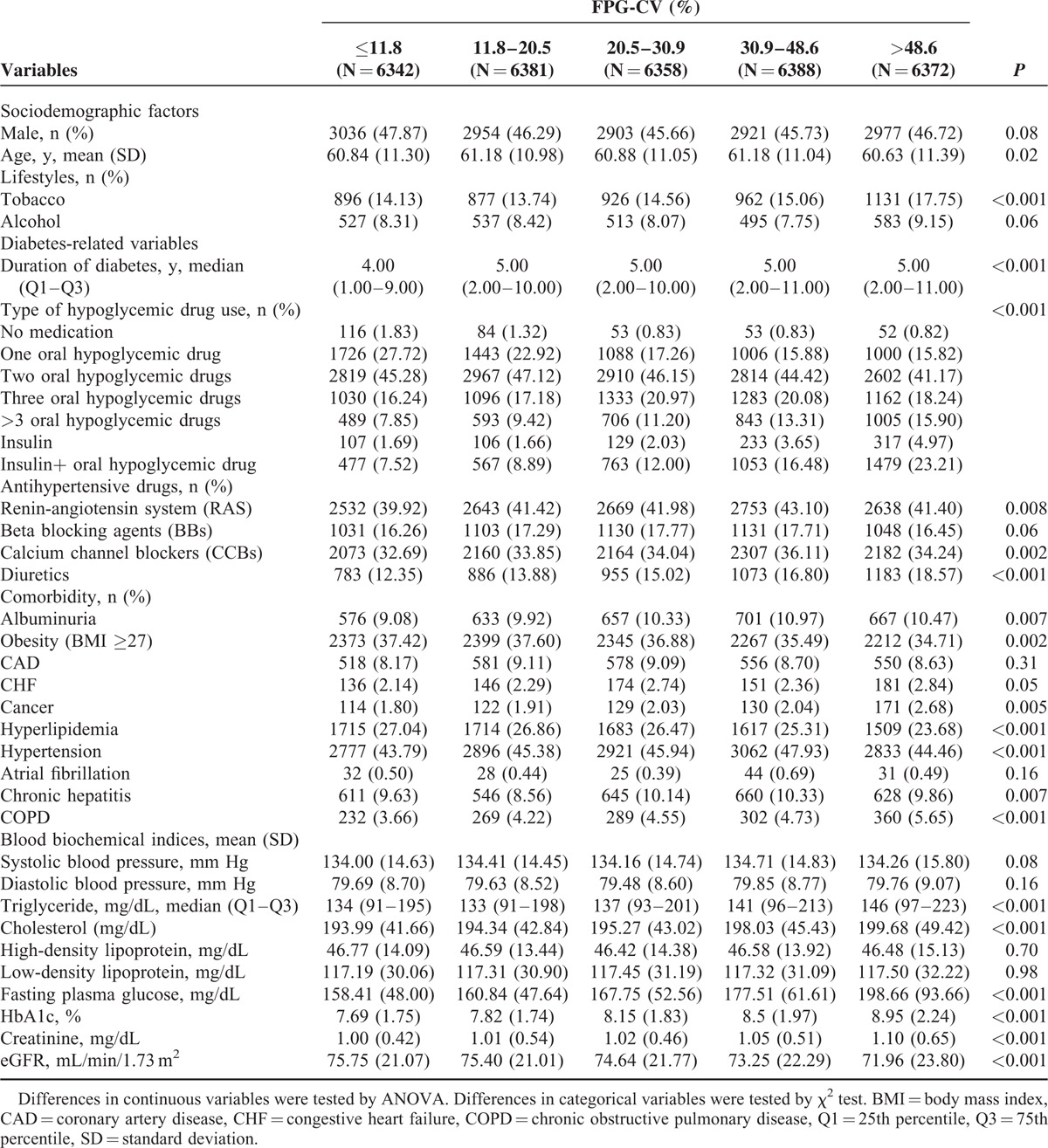
Comparisons of Baseline Sociodemographic Factors, Lifestyle Behaviors, Diabetes-Related Variables, Drug-Related Variables, and Comorbidity According to Quintile of FPG-CV in Patients With Type 2 Diabetes Enrolled in the NDCMP, Taiwan (n = 31,841)

Multivariate adjusted Cox regression was performed to examine the relative contribution of FPG-CV and HbA_1c_-CV to the development of ESRD. Table 3 shows HRs for ESRD in subjects grouped by quintiles of FPG-CV and HbA_1c_-CV. Both FPG-CV and HbA1c-CV were independent predictors for ESRD with age and sex adjustment. Considering mean values of the first year follow-up of fasting glucose and HbA_1c_, lifestyles, comorbidity, and complications, FPG-CV and HbA_1c_ effects were slightly attenuated but still remained significant. The correlation coefficient between FPG-CV and HbA_1c_-CV was determined to be 0.34 based on Pearson correlation, which was a weak correlation. These results showed no possibility of collinearity between FPG-CV and HbA_1c_-CV if both were simultaneously considered in the multivariate model. By simultaneously considering FPG-CV and HbA_1c_-CV in the model, both of them were independent predictors of ESRD with corresponding HR of 1.20 (95% CI 1.01, 1.41), 1.24 (95% CI 1.05, 1.46) in HbA_1c_-CV from fourth to fifth quintile and 1.13 (95% CI 0.95, 1.36), 1.23 (95% CI 1.03, 1.47) in FPG-CV. In addition, they were both independent predictors of all-cause mortality with corresponding HR of 1.15 (95% CI 1.06, 1.25), 1.23 (95% CI 1.13, 1.34) in HbA_1c_-CV from fourth to fifth quintile and 1.21 (95% CI 1.11, 1.31), 1.29 (95% CI 1.18, 1.40) in FPG-CV.

**TABLE 3:**
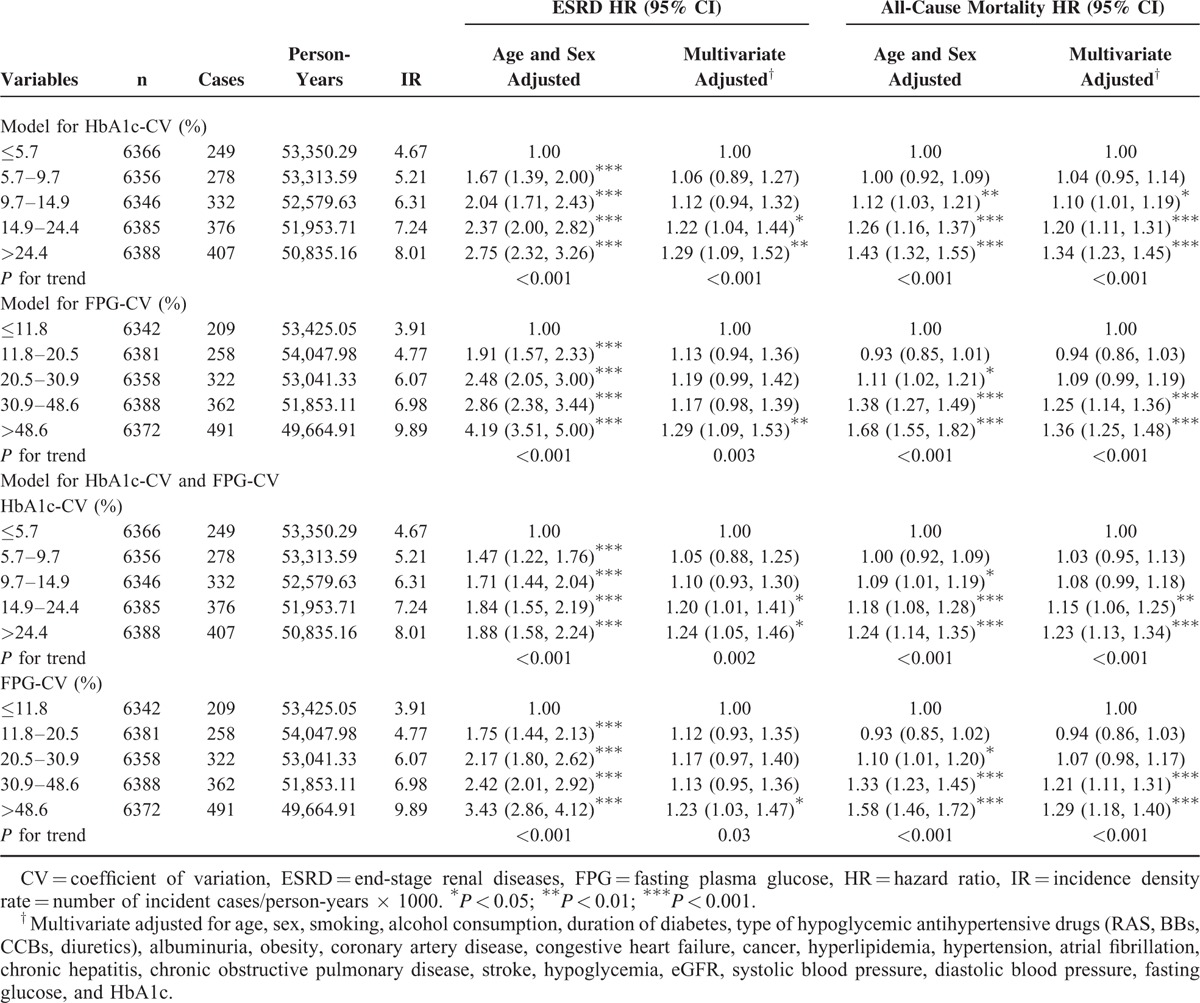
HRs of ESRD and All-Cause Mortality According to Different Quintiles of 1-Year HbA1c-CV and FPG-CV in patients With Diabetes Enrolled in the NDCMP, Taiwan

**FIGURE 2:**
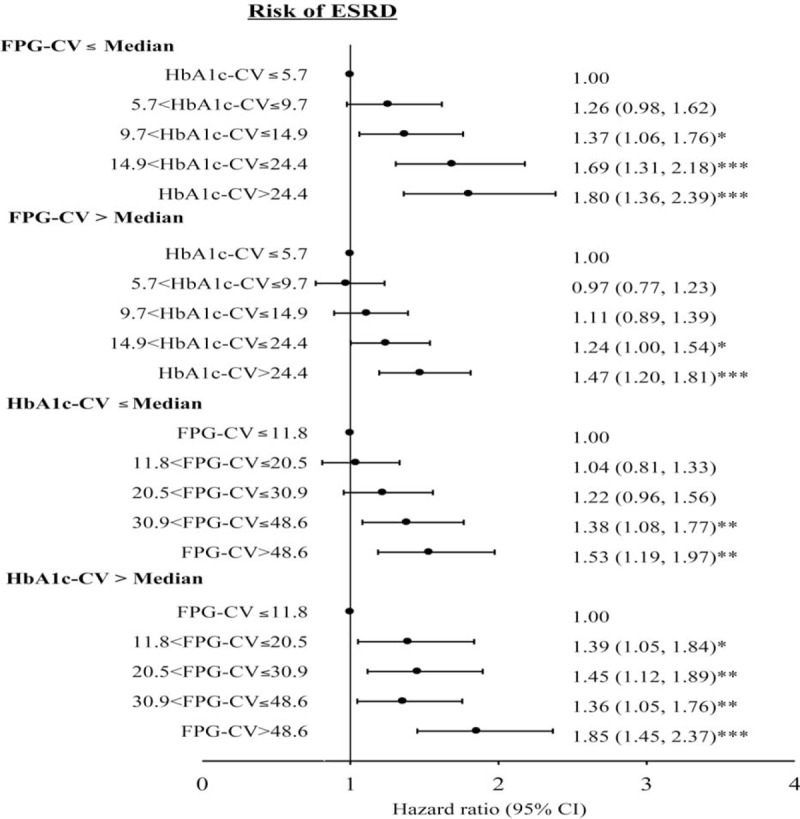
Risks of ESRD for HbA1C-CV stratified by FPG-CV (≤median or >median) and FPG-CV stratified by HbA1C-CV (≤median or >median) in patients with type 2 diabetes enrolled in the NDCMP, Taiwan. ^∗^*P* < 0.05; ^∗∗^*P* < 0.01; ^∗∗∗^*P* < 0.001. ESRD = end-stage renal diseases, NDCMP = National Diabetes Care Management Program.

To eliminate potential bias caused by the existence of comorbidities, sensitivity analyses were conducted, and patients with hyperglycemic hyperosmolar nonketotic coma, diabetic ketoacidosis, myocardial infarction, atrial fibrillation, and hypoglycemia were excluded (n = 1812). A similarly significant HRs for ESRD were observed among patients with a HbA_1c_-CV from fourth to fifth quintile (1.22 [95% CI 1.03, 1.45] and 1.28 [95% CI 1.08, 1.52]) and with a FPG-CV from fourth to fifth quintile (1.20 [95% CI 1.00, 1.44] and 1.30 [95% CI 1.09, 1.56]). The results of HbA_1c_-CV and FPG-CV, further stratification according to each other (Fig. 2), were generally consistent with those determined in the initial analysis. The interaction between HbA_1c_-CV and FPG-CV was statistically insignificant (*P* > 0.05). With HbA_1c_-CV subgrouped based on deciles, multivariate-adjusted HRs for HbA_1c_-CV levels from sixth to tenth decile were 1.36 (1.07, 1.74), 1.20 (0.94, 1.54), 1.37 (1.08, 1.75), 1.66 (1.32, 2.10), and 1.57 (1.23, 1.99); and for FPG-CV were 1.42 (1.11, 1.81), 1.26 (0.98, 1.61), 1.48 (1.16, 1.88), 1.82 (1.44, 2.29), and 1.77 (1.40, 2.24).

## DISCUSSION

We found that visit-to-visit glucose variations in both FPG-CV and HbA_1c_-CV independently predict ESRD in patients with type 2 diabetes >30 years. After adjusting for mean FPG, HbA_1c_, and other conventional risk factors, FPG-CV and HbA_1c_-CV still pinpoint the association between oscillating plasma glucose and ESRD. These findings are relevant to the clinical management of type 2 diabetes such that long-term CV of both FPG and HbA_1c_ should be evaluated, in addition to only reading the raw data. Recent therapies used should be evaluated for their potential to minimize glucose fluctuation in patients with type 2 diabetes to prevent ESRD.

Glucose variability has been measured in different ways,^17–20^ including with-in day blood glucose variation, hypoglycemia and hyperglycemia episodes, and visit-to-visit variation. Preferred measures of glycemic variability lack consensus. Previous studies that examine the effect of short-term intraday glucose variability on late diabetes complications in patients with type 1 diabetes have mixed results.^15,19,21^ However, long-term glycemic variance demonstrated a more consistent relationship with diabetes-related complications in patients with type 1 or type 2 diabetes.^11–13,22^ The spectra of FPG-CV, HbA_1c_-CV, and with-in day glucose variation might be different. Wide variation of FPG-CV and HbA_1c_-CV might reflect a more complicated clinical course, suboptimal medications, and self-management in addition to oxidative stress by acute variance of glucose.^13,23^ Although visit-to-visit glucose variation may not reflect actual glucose variation like self-monitored blood glucose (SMBG) or continuous monitor of glucose, it is difficult to long term measure SMBG or continuous blood glucose. The association of HbA_1c_ variation to proteinuria,^11^ chronic kidney disease (CKD),^13^ and ESRD,^13^ and FPG variation to DM nephropathy,^12^ had been reported. We fill the gap of FPG variation to ESRD. These findings provide evidence that these longer-term indexes of glucose variation might be proper indicators for estimating diabetic complications.

Patients with high glucose level may be subject to change of hypoglycemic drug use or more intensive diabetes control, that would lead to decreasing glucose level and higher variation in fasting plasma glucose. In order to rule out this possibility, we have examined change in type of hypoglycemic drug use between 1 year before index date and 1 year after index date, and the concordance of each type of hypoglycemic drug use was high, ranging from 94.91% to 99.13%. Thus, the possibility that change of hypoglycemic drug use or intensification of treatment that may explain the association between glycemic variation and ESRD would be less likely. In addition, we also evaluated the concordance of change in type of antihypertensive drug use, and the concordance rate was all above 95% for each type of medication use. Thus, the probability that changes in antihypertensive and/or hypoglycaemic medications may influence the effects of HbA1c-CV and FBG-CV on risk of ESRD was low.

Our data showed both FPG-CV and HbA_1c_-CV predict ESRD in patients with type 2 DM. Weak association of FPG-CV and HbA_1c_-CV is observed (*r* = 0.34, *P* < 0.001). Therefore, these 2 measures capture different aspects of glycemic variation. HbA_1c_ is regarded as mean glucose and does not reflect acute fluctuation in glucose level.^24^ The contribution of fasting or postprandial glucose varies with different HbA_1c_ levels while postprandial glucose contributed more at lower HbA_1c_ levels.^25,26^ HbA_1c_ is also affected by test time,^24^ elevated blood urea nitrogen level, metabolic acidosis, anemia, blood transfusion, hemoglobinopathies, erythropoietin-stimulating agents, and protein-energy wasting,^27^ which might occur in CKD patients. On the contrary, FPG level captures acute fluctuation in glucose level caused by irregular eating or lifestyle episode, which was not easily detected by HbA_1c_. Thus, FPG might be a more sensitive indicator than HbA_1c_ for capturing variation of glucose because of overindulgence in food given that HbA_1c_ is considered a mean. Extending FPG and HbA_1c_ to visit-to-visit FPG-CV and HbA_1c_-CV as a marker for glucose variability has 2 benefits. First, FPG and HbA_1c_ are routinely measured in almost all patients with diabetes, and estimating their CV is easy. Second, such approach seems to provide longer observation spectrum and good prediction power to further survival,^10,28^ cardiovascular events,^10^ stroke,^29^ DM nephropathy,^11,12,30^ and ESRD.^13^

Several mechanisms explained the impact of high glucose variation and renal toxicity, including increase in glomerular permeability,^31^ mesangial lipid accumulation,^32^ mesangial and tubulointerstitial cell matrix production,^33^ expression of fibrinogenesis markers, circulating level of inflammatory cytokines,^34^ endothelial dysfunction,^34^ and free radicals that activate the pathogenesis of diabetic complications.^35,36^

This study has limitations. First, it is an observational study, and a few residual and unrecognized confounding variables such as diet might be present. Thus, it is likely that a reduced risk of ESRD might reflect a delay of initiation of dialysis due to amelioration of uremic symptoms possibly due to diet rather than an actual slowing of the decline in renal function. Second, we only have 1-year FPG and HbA_1c_ measurements in NDCMP data set. Thus, we could not evaluate the intraindividual variation of FPG-CV and HbA_1c_-CV during follow-up and their dynamic effects on ESRD incidence. Third, FPG cannot possibly represent the actual glycemic level because of blood drawing without fasting, overeating, or gluttony before blood drawing because of social events and these factors cannot represent the dietary behavior of a typical day or dietary restriction before regular outpatient visits. These conditions would further result in measurement error for visit-to-visit variation for FPG represented by FPG-CV. However, these conditions would result in a consistent distortion of FPG-CV measurements to higher values. If a true association exists between FPG-CV and ESRD incidence, then this type of error would result in underestimation of the effect. Fourth, the present study did not measure postprandial glucose. The effect of postprandial hyperglycemia contributing to ESRD could not be assessed.

## CONCLUSIONS

In this large cohort of Chinese patients with type 2 diabetes, both FPG-CV and HbA1c-CV were independent predictors of ESRD, after adjusting mean FPG, HbA1c, and other conventional risk factors. Our results expand existing knowledge about the relationship of glycemia variation and ESRD, suggesting glucose variation using FPG-CV and HbA_1c_-CV might be used in managing patients with diabetes in predicting clinical prognosis. Future studies should assess the effects of interventions for reducing glucose variation on progression of diabetic nephropathy or ESRD incidence.
